# PET radioligand binding to translocator protein (TSPO) is increased in unmedicated depressed subjects

**DOI:** 10.1186/s13550-018-0401-9

**Published:** 2018-07-03

**Authors:** Erica M. Richards, Paolo Zanotti-Fregonara, Masahiro Fujita, Laura Newman, Cristan Farmer, Elizabeth D. Ballard, Rodrigo Machado-Vieira, Peixiong Yuan, Mark J. Niciu, Chul Hyoung Lyoo, Ioline D. Henter, Giacomo Salvadore, Wayne C. Drevets, Hartmuth Kolb, Robert B. Innis, Carlos A. Zarate Jr

**Affiliations:** 1 Intramural Research Program, National Institute of Mental Health, National Institutes of Health, Building 10, CRC Room 6-5340, 10 Center Drive, Bethesda, MD 20892 USA; 2 Houston Methodist Research Institute, Weill Cornell Medicine, Houston, Texas USA; 3 Department of Psychiatry and Behavioral Sciences, University of Texas Health Science Center, Houston, TX USA; 4 Department of Neurology, Gangnam Severance Hospital, Yonsei University College of Medicine, Seoul, South Korea; 5 Janssen Research and Development, LLC, Titusville, NJ USA

**Keywords:** Inflammation, Major depressive disorder, Biomarkers, Peripheral benzodiazepine receptor, Positron emission tomography

## Abstract

**Background:**

Inflammation is associated with major depressive disorder (MDD). Translocator protein 18 kDa (TSPO), a putative biomarker of neuroinflammation, is quantified using positron emission tomography (PET) and ^11^C-PBR28, a TSPO tracer. We sought to (1) investigate TSPO binding in MDD subjects currently experiencing a major depressive episode, (2) investigate the effects of antidepressants on TSPO binding, and (3) determine the relationship of peripheral and central inflammatory markers to cerebral TSPO binding. Twenty-eight depressed MDD subjects (unmedicated (*n* = 12) or medicated (*n* = 16)) and 20 healthy controls (HC) underwent PET imaging using ^11^C-PBR28. Total distribution volume (*V*_T_, proportional to Bmax/Kd) was measured and corrected with the free fraction in plasma (*fp*). The subgenual prefrontal cortex (sgPFC) and anterior cingulate cortex (ACC) were the primary regions of interest. Peripheral blood samples and cerebrospinal fluid were analyzed to investigate the relationship between TSPO binding and peripheral and central inflammatory markers, including interleukins and neurotrophic factors previously linked to depression.

**Results:**

TSPO binding was higher in MDD versus HC in the sgPFC (Cohen’s *d* = 0.64, *p* = .038, 95% CI 0.04–1.24) and ACC (*d* = 0.60, *p* = .049, 95% CI 0.001–1.21), though these comparisons missed the corrected threshold for statistical significance (*α* = .025). Exploratory analyses demonstrated that unmedicated MDD subjects had the highest level of TSPO binding, followed by medicated MDD subjects, who did not differ from HC. TSPO binding correlated with interleukin-5 in cerebrospinal fluid but with no other central inflammatory markers.

**Conclusions:**

This study found a trend towards increased TSPO binding in the brains of MDD subjects, and post hoc analysis extended these findings by demonstrating that this abnormality is significant in unmedicated (but not medicated) MDD subjects.

**Electronic supplementary material:**

The online version of this article (10.1186/s13550-018-0401-9) contains supplementary material, which is available to authorized users.

## Background

Inflammation is associated with major depressive disorder (MDD), both as a putative causal factor and as a biomarker of disease state and/or response to antidepressant treatment [1, 2]. Medical illnesses associated with peripheral inflammation, such as rheumatoid arthritis, systemic lupus erythematosus, and multiple sclerosis, have higher rates of comorbid MDD compared to the general population [3, 4]. Conversely, some pro-inflammatory markers are normalized in individuals with MDD treated with antidepressant medications or electroconvulsive therapy [5–8]. While most studies have linked MDD to increased peripheral inflammation, central inflammation—typically measured by evaluating inflammatory biomarkers in cerebrospinal fluid (CSF)—has also been associated with depression [9, 10], albeit less consistently.

Tumor necrosis factor-alpha (TNF-a), brain-derived neurotrophic factor (BDNF), C-reactive protein (CRP), and various interleukins (ILs)—including IL-5, IL-6, IL-8, and IL-10—have been implicated in the pathophysiology of MDD [5, 8, 11–15]. In some instances, however, the direction of differences relative to healthy controls have differed between peripheral and central levels of the same cytokine, making it difficult to extrapolate peripheral abnormalities to the brain [10, 16].

While differences in inflammatory markers in the blood and CSF are well-documented, it remains unclear whether inflammatory changes manifest in the brains of MDD subjects. Translocator protein 18 kDa (TSPO) is a mitochondrial protein that transports cholesterol to an enzyme that synthesizes pregnenolone, which is a precursor for steroids and neurosteroids [17]. Because TSPO is highly expressed in immune and glial cells within the brain—particularly activated microglia and reactive astrocytes—it is a potential biomarker of neuroinflammation and, furthermore, can be accurately quantified through PET [18]. A 2013 study found no differences in TSPO binding between healthy controls and MDD subjects [19]; however, a recent positron emission tomography (PET) study by Setiawan and colleagues found that, compared to healthy controls, subjects with MDD had increased TSPO density [20]. Using PET imaging and ^18^F-FEPPA, a radioligand for TSPO, the authors found a positive correlation between TSPO binding and severity of depression but observed no correlation between TSPO binding and several peripheral biomarkers of inflammation [20]. Thus, while neuroinflammation and MDD appeared to be linked in some studies, it remains unknown whether these findings are replicable, whether they may be influenced by antidepressant treatment, and whether peripheral markers of inflammation, such as TNF-alpha and other cytokines, correlate with TSPO density.

The primary aim of this study was to investigate TSPO binding in MDD subjects. The secondary aims were to determine if antidepressant use affects TSPO binding and whether TSPO binding is associated with peripheral and/or central biomarkers of inflammation. We conducted PET scans with medicated and unmedicated MDD subjects and healthy controls using ^11^C-PBR28—a TSPO tracer and a close chemical analog of ^18^F-FEPPA—as an indirect measure of neuroinflammation [21]. Both ^11^C-PBR28 and ^18^F-FEPPA are second-generation radioligands and, because TSPO is increased not only in activated microglia but also in reactive astrocytes, both ligands are considered able to indirectly measure neuroinflammation. We also sampled peripheral blood and cerebrospinal fluid to investigate the relationship between TSPO and inflammatory markers.

## Methods

### Participants

Participants included 28 subjects (10 women and 18 men, aged 18 or older) diagnosed with MDD, currently experiencing a moderate-to-severe major depressive episode (*n* = 12 unmedicated and *n* = 16 medicated, based on current state at intake) and 20 healthy controls with no current or prior mental health history (10 men and 10 women, aged 18 or older) (Table 1). Unmedicated subjects were medication-free for at least 2 weeks prior to the PET scan. All subjects were screened at the Clinical Research Center of the National Institute of Mental Health (NIMH) in Bethesda, Maryland, between January 2014 and March 2016 (ClinicalTrials.gov identifier: NCT01851356). Participants were diagnosed with MDD via an interview with a licensed, independent psychiatrist and confirmed with the Structured Clinical Interview for Axis I DSM-IV Disorders-Patient Version [22]. All subjects completed the Montgomery-Asberg Depression Rating Scale (MADRS) within 1 week prior to the PET scan; inclusion criteria required a score ≥ 20. Exclusion criteria were evidence of risk of imminent suicide, bipolar disorder, substance abuse within 6 months, or any comorbid illness likely to affect the subject’s inflammatory status. The additional exclusion criterion for healthy controls was any history of an Axis I diagnosis. All subjects were screened for the rs6971 polymorphism on the TSPO gene using in vitro TSPO phenotypic binding assays from leukocytes; six low-affinity binders (three MDD, three controls; rate did not differ between groups) were excluded from the study because they do not bind appreciable amounts of TSPO ligands [23].

**Table 1:** Demographic information

	Healthy controls		MDD-unmedicated		MDD-medicated			
	(*n* = 20)		(*n* = 12)		(*n* = 16)			
	Mean	SD	Mean	SD	Mean	SD	*F* (2,45)	*p*
Age	31.60	10.36	33.80	8.66	44.60	9.74	8.54	.001
BMI (kg/m^2^)	26.01	4.06	27.50	5.29	28.10	4.99	0.93	.40
Age of onset (years)	–	–	18.42	5.6	17.81	5.59	0.08	.78
Current episode (months)	–	–	40.67	59.55	57.44	92.86	0.30	.59
Length of illness (years)^a^	–	–	16.21	8.75	26.98	10.42	8.19	.008
MADRS total score	0.30	0.57	31.75	3.62	31.00	4.40	573.73	< .0001
	*n*	%	*n*	%	*n*	%	*X* ^2^	*p*
Sex (female)	10	50	5	42	6	38	0.59	.74
Race (Caucasian)	10	50	9	75	15	94	8.37	.02
Binding affinity (high)	12	60	10	83	9	56	2.51	.29

*MDD* major depressive disorder, *BMI* body mass index, *MADRS* Montgomery-Asberg Depression Rating Scale

^a^Missing data for *n* = 1 in MDD-medicated

This study was approved by the Combined Neuroscience Institutional Review Board of the National Institutes of Health. All participants gave written informed consent before entry into the study.

### ^11^C-PBR28 PET imaging

^11^C-PBR28 PET scans were performed on a GE Advance Tomograph (GE Medical Systems; Waukesha, WI). In two subjects, the images were instead acquired with a high-resolution research tomograph (Siemens Medical Solutions; Malvern, PA); images for these two subjects were reconstructed using the same resolution and parameters used for the GE scans. After a transmission scan for attenuation correction, ^11^C-PBR28 was injected in bolus (healthy volunteers, 701 ± 19 MBq; medicated MDD subjects, 694 ± 10 MBq; unmedicated MDD subjects, 700 ± 12 MBq), and the images were acquired in 3D for 90 min. Arterial blood samples were drawn manually at 15-s intervals for the first 2.5 min, then at 3, 4, 6, 8, 10, 15, 20, 30, 40, 50, 60, 75, and 90 min. Radioactivity in whole blood and plasma was measured by a gamma counter, and the parent concentration was obtained by high-performance liquid chromatography [24]. The free fraction of ^11^C-PBR28 in plasma (*f*_P_) was measured by ultrafiltration and normalized using a standard derived from pooled donor plasma to correct for day-to-day intra-assay variability [25] and yielded a value of 2.85 ± 0.76%, 2.98 ± 0.76%, and 2.49 ± 0.26%, respectively, for healthy volunteers, medicated, and unmedicated subjects with depression. *f*_P_ was not available for one healthy control subject, so the average group value from the other healthy controls was used.

All participants also underwent brain magnetic resonance imaging (MRI) within 1 year prior to the PET scan. T1-weighted MR images were acquired using a 3T Philips Achieva scanner (Bothell, WA) with turbo field echo sequence (repetition time = 8.1 ms, echo time = 3.7 ms, flip angle = 8, matrix = 181 × 256 × 256, voxel size = 1 × 0.983 × 0.983 mm).

PET brain time-activity curves were obtained with the Pneuro module of Pmod 3.8 (Zurich, Switzerland). Image pre-processing—such as coregistration between PET and MR, segmentation, and atlas normalization—was performed with the Pneuro pipeline. The regions of interest were automatically defined using the Hammers’ probabilistic brain atlas [26].

### Selection of brain regions

In order to reduce the likelihood of type II errors, we selected two regions of interest a priori: the anterior cingulate cortex (ACC) and the subgenual prefrontal cortex (sgPFC). These regions were selected because they have been shown to differ in individuals with MDD using multiple imaging modalities [27–30] and often normalize following antidepressant treatment [8]. Increased microglial quinolinic acid immunoreactivity in specific subregions of the ACC further underscores the involvement of these regions and supports the role of the immune system in the pathophysiology of MDD [31]. Seventy-five brain regions from both hemispheres—including the insula, putamen, thalamus, hippocampus, and cerebellum—were included in a secondary analysis to determine if changes in TSPO binding were more region-specific or globally distributed in the brain.

ACC and sgPFC values were obtained by averaging the left and right sides of each region. The sgPFC region was obtained by merging the subgenual, subcallosal, and presubgenual areas of the Hammers’ atlas.

### ^11^C-PBR28 PET imaging data analysis

At the regional level, total distribution volume (*V*_T_) was calculated using a two-tissue compartmental model and a metabolite-corrected arterial input function fitted to a tri-exponential function. Brain data were weighted frame-wise by assuming that the inverse square root of noise-equivalent counts was proportional to the normalized standard deviation of each frame. The arterial whole blood curve was used to correct for activity in the vascular component, assuming that the blood volume is 5% of the total brain volume. The delay between the arrival of the radioligand to the brain and to the radial artery was estimated by fitting the whole brain curve. *V*_T_ values were then corrected for *f*_P_ to obtain the final outcome parameter *V*_T_/*f*_P_. Uncorrected *V*_T_ values were also compared to test the robustness of these findings. Genotype (mixed-affinity vs high-affinity) was added as a covariate to all models.

At the voxel level, we calculated first the parametric images with the Logan plot and divided each image by the individual free fraction. The *V*_T_/*f*_P_ images of the three groups were then normalized and smoothed with an 8-mm Gaussian filter. Using SPM12 (Wellcome Trust, London, UK), an ANCOVA model was used for the comparison between the groups with the diagnosis and the genotype status as fixed factors and age and BMI as covariates. We first created significance map with cutoff *p* < 0.005 uncorrected for multiple comparisons and then applied cluster-wise correction for multiple comparisons with cluster-level cutoff *p* < 0.05.

### Peripheral and central biomarker analysis

All subjects provided peripheral blood samples, which were collected using the vacutainer system within 2 h before the PET scan. Blood samples were centrifuged at 3000 rpm at 4 °C for 10 min and stored at − 80 °C. For those who consented (*n* = 6 healthy controls, *n* = 4 unmedicated MDD, *n* = 10 medicated MDD), up to 15 mL CSF was collected within 1 week following the PET scan and immediately placed in liquid nitrogen and stored at − 80 °C.

The following peripheral and central markers of inflammation were examined as correlates of TSPO binding (see Additional file 1 for specific methods): vascular endothelial growth factor (VEGF), IL-6, IL-8, amyloid A1, adiponectin, BDNF (plasma only, not detected in CSF), TNF-alpha (plasma only), CRP (plasma only), IL-2 (CSF only, not detected in plasma), IL-5 (CSF only), and interferon-gamma (CSF only).

### Statistical analysis

The general linear model was used to evaluate the primary hypothesis, which was that TSPO binding is elevated among patients with MDD relative to HC, and the secondary hypothesis, which was that antidepressant use among MDD patients, would be associated with normalized TSPO binding. Genotype was entered as a covariate; no other covariates were specified a priori (see Additional file 1 for additional information). Partial Pearson correlations were used to evaluate the relationship between TSPO binding and inflammatory markers; body mass index (BMI) was added as a covariate in these analyses. Alpha was set to .025 per region (ACC and sgPFC) for the primary aim, and alpha for the secondary and exploratory aims was unadjusted (.05). Cohen’s *d* effect sizes (with 95% CI) were calculated using least square mean difference estimates and associated degrees of freedom. Data were analyzed using SAS/STAT Version 9.4.

## Results

Demographic information is presented in Table 1. Most (*n* = 15) subjects in the medicated MDD group were on multiple psychoactive medications (median = 2.5) including selective serotonin reuptake inhibitors, serotonin-norepinephrine reuptake inhibitors, serotonin modulators, tricyclic antidepressants, and/or other psychiatric medications (see Additional file 1 for additional information on concomitant medications).

**Fig. 1:**
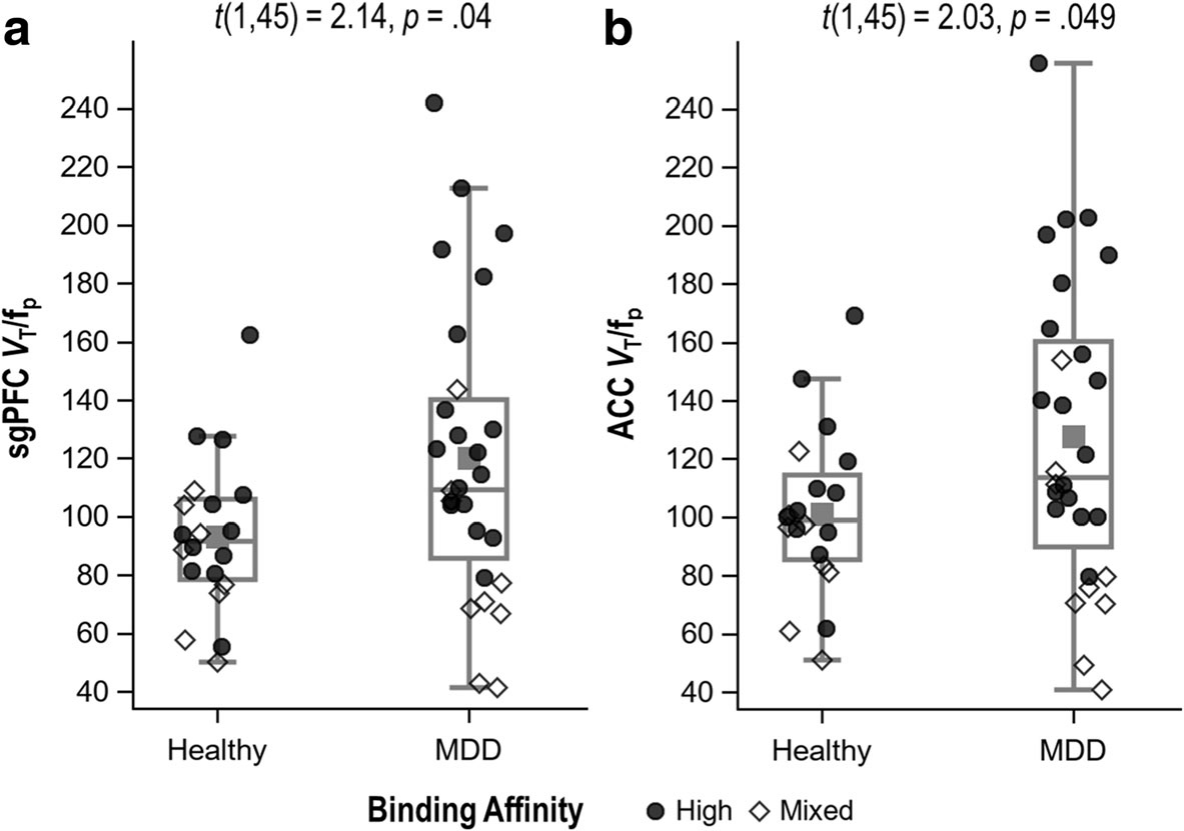
Translocator protein (TSPO) binding in the subgenual prefrontal cortex (sgPFC) (**a**) and anterior cingulate cortex (ACC) (**b**) in individuals with a major depressive disorder (MDD) (*n* = 28) and healthy controls (*n* = 20). Groups are compared using ANCOVA, controlling for genotype

**Fig. 2:**
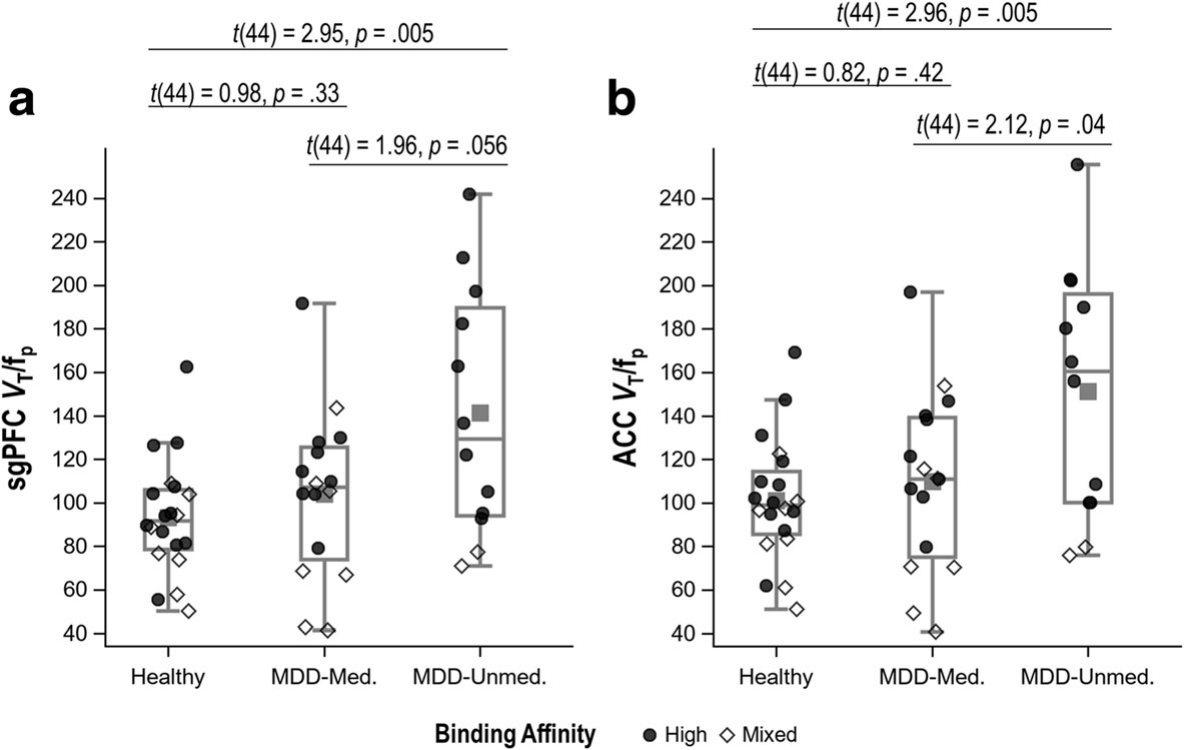
Translocator protein (TSPO) binding in the subgenual prefrontal cortex (sgPFC) (**a**) and anterior cingulate cortex (ACC) (**b**) among healthy controls and medicated and unmedicated patients with a major depressive disorder (MDD). Groups are compared using ANCOVA, controlling for genotype

There was a trend towards higher TSPO binding in MDD versus HC (sgPFC: Cohen’s *d* = 0.64, *p* = .038, 95% CI 0.04–1.24; ACC: *d* = 0.60, *p* = .049, 95% CI 0.001–1.21), though these comparisons missed the corrected significance threshold (*α* = .025) (see Fig. 1, Additional file 1: Table S1). In secondary analyses, TSPO binding was highest in unmedicated MDD participants, followed by medicated MDD participants and HC, which did not differ from one another (see Fig. 2, Additional file 1: Table S2). For both sgPFC and ACC, the differences between unmedicated MDD participants and HC were large (in both regions *p* = .005, *d* = 0.89, 95% CI 0.28–1.50) while the differences between unmedicated and medicated MDD participants were moderate (sgPFC: *p* = .056, *d* = 0.59, 95% CI − 0.02–1.20; ACC: *p* = .04, *d* = 0.64, 95% CI 0.03–1.25).

This pattern of results was consistent across most regions of interest (see Additional file 1: Tables S3 and S4). Sensitivity analyses using uncorrected *V*_T_ yielded consistent results for MDD versus HC, but all between-group comparisons among unmedicated MDD, medicated MDD, and HC were non-significant (see Additional file 1: Table S5).

**Fig. 3:**
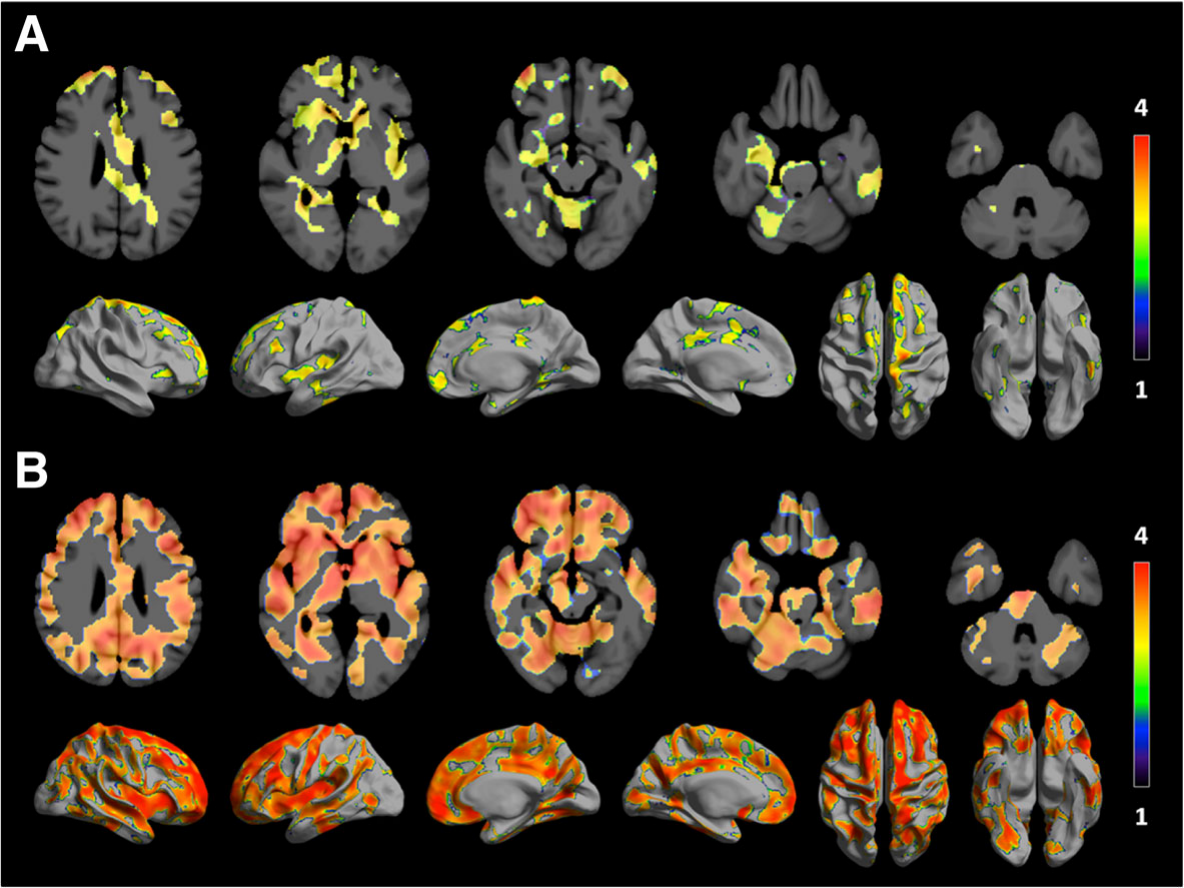
Statistical significance maps rendered on axial slices and semi-inflated surface showing increase in ^11^C-PBR28 binding in unmedicated depressed patients compared to healthy controls (**a**). **b** The same analysis is restricted to high-affinity binder subjects, done with the more stringent threshold of *p* < 0.001. Only clusters with threshold *p* < 0.05 for cluster-wise multiple comparisons are displayed. The color bar represents *T* values. The same comparison using only mixed-affinity binders yielded no significant differences, but the unmedicated group comprised only two mixed-affinity binders

Similar to region analysis, the voxel-wise comparison showed higher ^11^C-PBR28 binding across several regions of the brain of unmedicated MDD subjects compared to healthy controls. Areas of significant difference, after correction for multiple comparisons, were spread mostly across the frontal and temporal lobes, bilaterally (Fig. 3). Even for the liberal uncorrected threshold of *p* < 0.005, no cluster survived the correction for multiple comparisons when calculating the difference between medicated subjects and healthy controls and the difference between medicated and unmedicated subjects.

We explored whether any particular drug class was associated with decreased binding. Among the total MDD group, the number of drug classes prescribed was not related to TSPO binding (*p* = .40). No individual drug class was associated with TSPO binding (all *p* > .10). However, because the number of participants prescribed any given drug class was small (see Additional file 1), the power to detect an effect of a given class was low.

No covariates in addition to genotype were specified a priori. However, differences between groups in age and race were observed (see Table 1). Further, a recent study from our laboratory suggested that age is associated with increased TSPO binding in healthy subjects [32], and at least one prior study documented a negative and moderately sized correlation between BMI and TSPO *V*_T_ in select brain regions [20]. For these reasons, analyses including age, race, and BMI as additional covariates were performed.

The addition of covariates did not alter the interpretation of the MDD versus HC analyses (see Additional file 1: Table S1). However, the introduction of additional covariates strengthened the differences between the medicated and unmedicated MDD patients, and between the unmedicated MDD patients and HC, while the medicated MDD patients remained undifferentiated from HC (see Additional file 1: Table S2).

Length of illness in MDD subjects was not related to TSPO binding (sgPFC: *r* = .15, *p* = .46; ACC: *r* = .13, *p* = .52), and in MDD, TSPO binding did not correlate with MADRS score (sgPFC: *r* = − .09, *p* = .65; ACC: *r* = − .11, *p* = .56). After controlling for BMI, there were no significant between-group (MDD vs HC) differences in inflammatory marker concentrations (data not shown). Only plasma adiponectin and CSF IL-5 were found to be correlated with TSPO binding (Table 2).

**Table 2:** Partial Pearson correlations, controlling for body mass index, between TSPO binding and central and peripheral markers of inflammation

	Plasma (*n* = 48)		Cerebrospinal fluid (*n* = 20)	
	sgPFC	ACC	sgPFC	ACC
Interleukin 2	**–**	–	0.01	−0.04
Interleukin 5	**–**	–	− 0.59*	− 0.53*
Interleukin 6	− 0.16	− 0.15	− 0.25	−0.26
Interleukin 8	0.09	0.12	−0.19	−0.23
Brain-derived neurotrophic factor	0.25	0.23	**–**	–
Vascular endothelial growth factor	0.16	0.20	−0.43	−0.39
Interferon-gamma	**–**	–	− 0.39	−0.30
Tumor necrosis factor alpha	−0.01	0.02	**–**	–
C-reactive protein	−0.07	−0.08	**–**	–
Amyloid A1	− 0.23	−0.23	− 0.40	−0.43
Adiponectin	0.29*	0.29*	0.01	0.01

*sgPFC* subgenual prefrontal cortex, *ACC* anterior cingulate cortex, – data not available

*p ≤ .05

## Discussion

We found increased TSPO binding in the ACC and sgPFC (which were the two regions specified a priori) and throughout the brain of subjects with MDD replicating recent findings [20]. Because increased TSPO binding is an indirect marker of neuroinflammation, these results collectively provide evidence that neuroinflammation is increased in unmedicated MDD subjects and support brain inflammation as a therapeutic target for novel medication interventions. These findings contrast with a prior study that found no difference in TSPO binding between MDD subjects and healthy controls [19]; however, our study differs from that one in that we had a larger sample (*n* = 48 vs 22), with depression that was more severe (average MADRS = 31.5 vs 19.7) and current (in the previous study, some MDD participants had no current symptoms). We also observed no correlation with MADRS ratings, suggesting that TSPO is not a marker of disease severity in this subject population.

Consistent with the work of Setiawan and colleagues [20], two additional studies demonstrated increased TSPO binding in the brain of patients with MDD [33, 34]. This finding is true when different TSPO radioligands are used (^11^C-PBR28 vs ^18^F-FEPPA). A fourth, smaller (*n* = 10) study documented *decreased* TSPO binding in MDD, but that study excluded patients with elevated plasma concentrations of C-reactive protein, which may be the subset most likely to show increased TSPO in the brain [19]. Thus, this study is the fourth of five to find increased TSPO binding in MDD.

We found that TSPO binding in unmedicated MDD, but not medicated MDD, differed from healthy controls. This suggests that antidepressant treatment itself might normalize TSPO density, even without resolving depressive symptoms. Antidepressant medications have been shown to decrease levels of several peripheral inflammatory markers, including CRP and IL-6 [35, 36]. It is also possible that some SSRIs may decrease microglial activation by inhibiting elevations in intracellular calcium [37]. An experimental study is required to definitively address this question.

We note that although the TSPO density of the medicated MDD did not differ from healthy controls, these subjects were still depressed. This suggests that any anti-inflammatory effects of antidepressant medication, which are thought to act via indirect effects on the inflammatory system, are not sufficient for antidepressant response, though they may still be an important target in a subset of patients. The heterogeneity of MDD highlights the importance of identifying subgroups that may respond to specific interventions. Similar to other studies where subject enrichment helped elucidate specific subgroups who may respond to particular antidepressant interventions (e.g., elevated CRP in studies of inflammation and depression) [38], administering anti-inflammatory treatment interventions to MDD subjects selected for increased TSPO binding may be key to future studies.

Finally, we found that TSPO binding in the sgPFC and ACC correlated significantly and moderately with CSF IL-5 levels; however, no correlation with other peripheral or central markers of inflammation was observed. Overall, no difference in baseline levels of peripheral inflammation was observed between groups, indicating that TSPO binding is not simply a manifestation of changes in underlying baseline peripheral inflammation. These findings suggest that we have yet to identify a specific peripheral blood-based biomarker that may help us understand or predict the inflammatory changes that occur in the brains of subjects experiencing a major depressive episode. Nevertheless, the findings also suggest that additional investigation into IL-5—a key mediator of eosinophil activation—is needed. Some studies have implicated IL-5 in major depressive episodes [5, 39], though others found no significant difference in IL-5 levels between MDD subjects and healthy controls [3]. Our inability to identify biomarkers in CSF or plasma that correlate with TSPO binding likely indicates two key issues, namely, the heterogeneity of the underlying factors contributing to MDD and the limitations of currently available methods for detecting very small changes in blood/CSF biomarker concentrations. Notably, other cytokines and neurotrophic factors have been associated with psychiatric illness and may warrant further investigation. Examples include IL-1 and TGF, which have been implicated in the pathophysiology of multiple psychiatric disorders [40, 41].

Despite these interesting findings, the study has several limitations. First, although increased TSPO has been found in several CNS disorders with localized or widespread neuroinflammation, its cellular localization is not specific activated microglia, which are arguably viewed as the major source of cytokines in the brain. For example, TSPO is also found in astrocytes, including especially high densities in astroglyosis or scars that appear to have no currently active inflammatory processes [42]. In addition, immunohistochemical studies have found that TSPO is associated with vascular endothelium [43]. Although immunohistochemistry lacks the resolution to separate vascular endothelium from the immediately adjacent foot processes of astrocytes, this component of total TSPO binding is certainly not microglial in origin.

## Conclusions

In summary, the relative distribution of TSPO among cells of the brain is unknown, and the increased uptake measured in large regions of the brain with PET and their differing roles, related or unrelated to neuroinflammation, are yet to be clarified. Second, measuring *V*_T_/*f*p requires frequent arterial sampling from the subject. While PET studies using TSPO ligands in other disease states (e.g., Alzheimer’s disease) have reference regions that may be used instead of arterial sampling [44], the universal increases in TSPO binding observed in unmedicated MDD subjects indicate that no reference region exists in the brain and that arterial sampling will be required to measure differences between patients and controls.

Finally, it should be noted that in the field of mental health, replication studies are rare—largely due to the heterogeneity of patients, relatively small sample sizes available for research studies, and slight differences in experimental design—and the same is true in PET imaging studies. Such research ensures that future planned studies are as informed as possible and allow us, as a field, to move forward in our search to understand the pathophysiology of MDD and develop potential treatments for this devastating disorder. Regarding the present findings, future work may pool the data from this study with others to increase power and generalizability.

In conclusion, TSPO binding is increased in the brains of MDD subjects, and this appears to be diminished with antidepressant treatment. This indirect measurement of neuroinflammation during major depressive episodes suggests that inflammation should continue to be a focus in developing novel therapeutics for the treatment of MDD.

## Additional file

Additional file 1: **Table S1.** Results of ANCOVAs comparing TSPO binding (*V*_T_/*f*_P_) in MDD patients to healthy controls (HC). Table S2. Results of ANCOVAs comparing TSPO binding (*V*_T_/*f*_P_) in MDD patients with and without antidepressant medication to healthy controls (HC). Table S3 Results of ANCOVAs comparing TSPO binding (*V*_T_/*f*_P_) in MDD patients to healthy controls (HC) in additional representative regions of interest from the right hemisphere. Table S4. Results of ANCOVAs comparing TSPO binding (*V*_T_/*f*_P_) in healthy controls (HC) to medicated and unmedicated patients with MDD in additional representative regions of interest from the right hemisphere. Table S5. Results of sensitivity analyses using *V*_T_. (DOCX 43 kb)
