# Supplementary material for: PET radioligand binding to translocator protein (TSPO) is increased in unmedicated depressed subjects
Source: EJNMMI Res. 2018 Jul 3;8:57. doi: 10.1186/s13550-018-0401-9 (PMC6029989; doi:10.1186/s13550-018-0401-9)
Supplement: Supplementary file 1 — Table S1. Results of ANCOVAs comparing TSPO binding (VT/fP) in MDD patients to healthy controls (HC). Table S2. Results of ANCOVAs comparing TSPO binding (VT/fP) in MDD patients with and without antidepressant medication to healthy controls (HC). Table S3 Results of ANCOVAs comparing TSPO binding (VT/fP) in MDD patients to healthy controls (HC) in additional representative regions of interest from the right hemisphere. Table S4. Results of ANCOVAs comparing TSPO binding (VT/fP) in healthy controls (HC) to medicated and unmedicated patients with MDD in additional representative regions of interest from the right hemisphere. Table S5. Results of sensitivity analyses using VT. (DOCX 43 kb) [file 13550_2018_401_MOESM1_ESM.docx]

**Quantification of peripheral and central markers of inflammation.** When ready for assay, the ChemiKine BDNF (Millipore, MA, USA) or Quantikine VEGF (R&D Systems, MN, USA) sandwich ELISA kits were used according to the manufacturer's instructions. For BDNF, plasma was diluted 1:5 with a sample diluent, and the assay was performed in duplicate. In order to create the standard curves for BDNF and VEGF levels, a standard solution was diluted according to the manufacturer’s instructions. Biotin-Antibody (for BDNF) or Streptavidin-HRP (for VEGF conjugate) substrate and stop solution were added, and concentrations were then determined by absorbance at 450 nm using optical density values based on the standard curve values. CRP values were obtained from routine lab sampling, then determined by the laboratory services of the NIH Clinical Center. Multiplex arrays were similarly used to quantify IL-2, IL-5, IL-6, IL-8, TNF-alpha, amyloid A1, interferon-gamma, and adiponectin concentrations similar to methods used by Machado-Vieira and colleagues ([1](#_ENREF_1)). High sensitivity multiplex Luminex immunoassay (xMAP technology) and the fluorescently color-coded magnetic microsphere beads from R&D Systems (Minneapolis, MN) were used according to the manufacturer’s instructions. After the addition of biotinylated antibody cocktail and streptavidin-PE, levels of all analytes were determined using a Bio-Plex Magpix Multiplex Reader (Bio-Rad, CA). Concentration values were calculated automatically with Bio-Plex Manager MP Software by generating a five parameter logistic curve-fit standard curve.

**Statistical analysis.** Initial inspection of the data suggested that the homogeneity of variance assumption was violated. However, the general linear model is robust to this violation as long as group sizes are equal (ratio of <1.5), so we proceeded with standard ANCOVAs; results of models where variances were estimated separately by group and degrees of freedom were corrected using the Satterthwaite approximation and are available from the authors. The assumption of normality was assessed and satisfied via visual inspection of residual plots. Sensitivity analyses were performed using *V*_T_ with genotype entered as a covariate.

**Results.** Most (n=15) members of the medicated MMD group were on multiple psychoactive medications (median =2.5). The most common classes were selective serotonin reuptake inhibitors (n=8), serotonin-norepinephrine reuptake inhibitors (n=7), and benzodiazepines (n=6). Three participants took tricyclic antidepressants and two participants took serotonin antagonist and reuptake inhibitors. Two participants took lithium, three took antipsychotics, one took an anticonvulsant, and three took an amphetamine.

1. Machado-Vieira R, Gold PW, Luckenbaugh DA, Ballard ED, Richards EM, Henter ID, et al. (2017): The role of adipokines in the rapid antidepressant effects of ketamine. *Mol Psychiatry*. 22:127-133.

**Table S1. Results of ANCOVAs comparing TSPO binding (*V*_T_/*f*_P_) in MDD patients to healthy controls (HC)**

| **Effect** | **B** | **SE** | **DF** | **t** | **p** | **Cohen's d** | **95% CI** |
| --- | --- | --- | --- | --- | --- | --- | --- |
| **Primary Analyses** | | | | | | | |
| **Subgenual Prefontal Cortex (sgPFC)** |  |  |  |  |  |  |  |
| Intercept | 92.30 | 10.40 | 45 | 8.88 | <.0001 |  |  |
| Genotype High-Affinity (vs. Mixed) | 40.85 | 11.28 | 45 | 3.62 | .001 |  |  |
| Healthy (vs. MDD) | -23.44 | 10.94 | 45 | -2.14 | .038 | -0.64 | -1.24 – -0.04 |
| **Anterior Cingulate Cortex (ACC)** |  |  |  |  |  |  |  |
| Intercept | 96.85 | 10.73 | 45 | 9.03 | <.0001 |  |  |
| Genotype High-Affinity (vs. Mixed) | 45.39 | 11.64 | 45 | 3.90 | .0001 |  |  |
| Healthy (vs. MDD) | -22.89 | 11.29 | 45 | -2.03 | .049 | -0.60 | -1.21 – -0.002 |
| **Secondary Analyses (with additional covariates)** | | | | | | | |
| **Subgenual Prefontal Cortex (sgPFC)** |  |  |  |  |  |  |  |
| Intercept | 161.33 | 38.10 | 42 | 4.23 | .000 |  |  |
| Age | 0.57 | 0.49 | 42 | 1.16 | .254 |  |  |
| BMI | -3.36 | 1.12 | 42 | -2.99 | .005 |  |  |
| Non-White (vs. White) | -6.58 | 12.43 | 42 | -0.53 | .600 |  |  |
| Genotype High-Affinity (vs. Mixed) | 45.08 | 10.64 | 42 | 4.24 | .0001 |  |  |
| Healthy (vs. MDD) | -22.15 | 11.65 | 42 | -1.90 | .064 | -0.59 | -1.21 – 0.04 |
| **Anterior Cingulate Cortex (ACC)** |  |  |  |  |  |  |  |
| Intercept | 178.07 | 38.38 | 42 | 4.64 | <.0001 |  |  |
| Age | 0.58 | 0.49 | 42 | 1.17 | .250 |  |  |
| BMI | -3.83 | 1.13 | 42 | -3.38 | .002 |  |  |
| Non-White (vs. White) | -2.78 | 12.52 | 42 | -0.22 | .825 |  |  |
| Genotype High-Affinity (vs. Mixed) | 49.53 | 10.72 | 42 | 4.62 | <.0001 |  |  |
| Healthy (vs. MDD) | -23.75 | 11.73 | 42 | -2.02 | .049 | -0.62 | -1.25 – -0.004 |

**Note:** No additional covariates were specified a priori. Demographic characteristics found to differ between groups (age, BMI, race) were entered as covariates in secondary analyses.

**Table S2. Results of ANCOVAs comparing TSPO binding (*V*_T_/*f*_P_) in MDD patients with and without antidepressant medication to healthy controls (HC)**

| **Effect** | **B** | **SE** | **DF** | **t** | **p** | **Cohen's d** | **95% CI** |
| --- | --- | --- | --- | --- | --- | --- | --- |
| **No Additional Covariates** | | | | | | | |
| **Subgenual Prefontal Cortex (sgPFC)** |  |  |  |  |  |  |  |
| Intercept | 111.35 | 13.99 | 44 | 7.96 | <.0001 |  |  |
| Genotype High-Affinity (vs. Mixed) | 36.13 | 11.20 | 44 | 3.23 | .002 |  |  |
| Healthy (vs. Unmedicated MDD) | -39.65 | 13.44 | 44 | -2.95 | .005 | -0.89 | -1.50 – -0.28 |
| Medicated MDD (vs. Unmedicated MDD) | -27.72 | 14.12 | 44 | -1.96 | .056 | -0.59 | -1.20 – 0.02 |
| Healthy (vs. Medicated MDD) | -11.93 | 12.12 | 44 | -0.98 | .330 | -0.30 | -0.90 – 0.31 |
| **Anterior Cingulate Cortex (ACC)** |  |  |  |  |  |  |  |
| Intercept | 117.95 | 14.34 | 44 | 8.22 | <.0001 |  |  |
| Genotype High-Affinity (vs. Mixed) | 40.16 | 11.48 | 44 | 3.50 | .001 |  |  |
| Healthy (vs. Unmedicated MDD) | -40.86 | 13.78 | 44 | -2.96 | .005 | -0.89 | -1.50 – -0.29 |
| Medicated MDD (vs. Unmedicated MDD) | -30.72 | 14.48 | 44 | -2.12 | .040 | -0.64 | -1.25 – -0.03 |
| Healthy (vs. Medicated MDD) | -10.14 | 12.43 | 44 | -0.82 | .419 | -0.25 | -0.85 – 0.36 |
| **With Covariates** | | | | | | | |
| **Subgenual Prefontal Cortex (sgPFC)** |  |  |  |  |  |  |  |
| Intercept | 163.54 | 35.31 | 41 | 4.63 | <.0001 |  |  |
| Age | 1.09 | 0.49 | 41 | 2.22 | .032 |  |  |
| BMI | -3.26 | 1.04 | 41 | -3.13 | .003 |  |  |
| Non-White (vs. White) | -9.35 | 11.56 | 41 | -0.81 | .423 |  |  |
| Genotype High-Affinity (vs. Mixed) | 39.65 | 10.04 | 41 | 3.95 | .000 |  |  |
| Healthy (vs. Unmedicated MDD) | -39.03 | 12.34 | 41 | -3.16 | .003 | -0.99 | -1.62 – -0.36 |
| Medicated MDD (vs. Unmedicated MDD) | -38.54 | 13.68 | 41 | -2.82 | .007 | -0.88 | -1.51 – -0.25 |
| Healthy (vs. Medicated MDD) | -0.49 | 13.25 | 41 | -0.04 | .971 | -0.01 | -0.64 – 0.62 |
| **Anterior Cingulate Cortex (ACC)** |  |  |  |  |  |  |  |
| Intercept | 180.41 | 35.15 | 41 | 5.13 | <.0001 |  |  |
| Age | 1.14 | 0.49 | 41 | 2.32 | .025 |  |  |
| BMI | -3.72 | 1.04 | 41 | -3.58 | .001 |  |  |
| Non-White (vs. White) | -5.74 | 11.51 | 41 | -0.50 | .621 |  |  |
| Genotype High-Affinity (vs. Mixed) | 43.75 | 10.00 | 41 | 4.38 | <.0001 |  |  |
| Healthy (vs. Unmedicated MDD) | -41.72 | 12.29 | 41 | -3.39 | .002 | -1.06 | -1.69 – -0.43 |
| Medicated MDD (vs. Unmedicated MDD) | -41.04 | 13.62 | 41 | -3.01 | .004 | -0.94 | -1.57 – -0.31 |
| Healthy (vs. Medicated MDD) | -0.68 | 13.19 | 41 | -0.05 | .959 | -0.02 | -0.65 – 0.61 |

**Note:** No additional covariates were specified a priori. Demographic characteristics found to differ between groups (age, BMI, race) were entered as covariates in secondary analyses.

**Table S3. Results of ANCOVAs comparing TSPO binding (*V*_T_/*f*_P_) in MDD patients to healthy controls (HC) in additional representative regions of interest from the right hemisphere**

| Region | B | SE | DF | t | p | FDR-adjusted p |
| --- | --- | --- | --- | --- | --- | --- |
| Frontal inferior | -23.21 | 11.12 | 45 | -2.09 | .043 | .110 |
| Parietal superior | -21.41 | 10.67 | 45 | -2.01 | .051 | .110 |
| Temporal posterior | -20.22 | 10.73 | 45 | -1.88 | .066 | .110 |
| Thalamus | -23.49 | 12.40 | 45 | -1.89 | .065 | .110 |

**Note:** Genotype was entered as a covariate in all analyses (not shown). Estimate (B) is difference between HC and MDD (HC-MDD). P-value was FDR adjusted for comparisons at all regions.

**Table S4. Results of ANCOVAs comparing TSPO binding (*V*_T_/*f*_P_) in healthy controls (HC) to medicated and unmedicated patients with MDD in additional representative regions of interest from the right hemisphere**

| Region | Group1 | Group2 | B | SE | DF | t | p | FDR-adjusted p |
| --- | --- | --- | --- | --- | --- | --- | --- | --- |
| Frontal inferior | Healthy | MDD-Med. | -10.97 | 12.27 | 44 | -0.89 | .376 | .496 |
|  | Healthy | MDD-Unmed. | -40.45 | 13.61 | 44 | -2.97 | .005 | .050 |
|  | MDD-Med. | MDD-Unmed. | -29.48 | 14.30 | 44 | -2.06 | .045 | .103 |
| Parietal superior | Healthy | MDD-Med. | -8.73 | 11.67 | 44 | -0.75 | .458 | .551 |
|  | Healthy | MDD-Unmed. | -39.25 | 12.95 | 44 | -3.03 | .004 | .050 |
|  | MDD-Med. | MDD-Unmed. | -30.52 | 13.60 | 44 | -2.24 | .030 | .090 |
| Temporal posterior | Healthy | MDD-Med. | -8.12 | 11.81 | 44 | -0.69 | .496 | .563 |
|  | Healthy | MDD-Unmed. | -37.26 | 13.10 | 44 | -2.84 | .007 | .050 |
|  | MDD-Med. | MDD-Unmed. | -29.14 | 13.76 | 44 | -2.12 | .040 | .098 |
| Thalamus | Healthy | MDD-Med. | -9.28 | 13.63 | 44 | -0.68 | .500 | .563 |
|  | Healthy | MDD-Unmed. | -43.51 | 15.12 | 44 | -2.88 | .006 | .050 |
|  | MDD-Med. | MDD-Unmed. | -34.23 | 15.88 | 44 | -2.16 | .037 | .097 |

**Note:** Genotype was entered as a covariate in all analyses (not shown). Estimate (B) is difference between Group 1 and Group 2 (Group 1 – Group 2). P-value was FDR adjusted for comparisons at all regions.

**Table S5. Results of sensitivity analyses using *V*_T_**

| **Effect** | **B** | **SE** | **DF** | **t** | **p** |
| --- | --- | --- | --- | --- | --- |
| **Sensitivity: Primary Analyses (No Additional Covariates)** | | | | | |
| **Subgenual Prefontal Cortex (sgPFC)** |  |  |  |  |  |
| Intercept | 2.41 | 0.33 | 45 | 7.35 | <.0001 |
| Genotype High-Affinity (vs. Mixed) | 1.33 | 0.36 | 45 | 3.73 | 0.001 |
| Healthy (vs. MDD) | -0.66 | 0.35 | 45 | -1.90 | 0.064 |
| **Anterior Cingulate Cortex (ACC)** |  |  |  |  |  |
| Intercept | 2.53 | 0.34 | 45 | 7.41 | <.0001 |
| Genotype High-Affinity (vs. Mixed) | 1.47 | 0.37 | 45 | 3.97 | 0.000 |
| Healthy (vs. MDD) | -0.65 | 0.36 | 45 | -1.81 | 0.078 |
| **Sensitivity: Secondary Analyses (with additional covariates)** | | | | | |
| **Subgenual Prefontal Cortex (sgPFC)** |  |  |  |  |  |
| Intercept | 4.82 | 1.11 | 42 | 4.34 | <.0001 |
| Age | 0.03 | 0.01 | 42 | 1.93 | 0.060 |
| BMI | -0.13 | 0.03 | 42 | -3.95 | 0.000 |
| Non-White (vs. White) | -0.08 | 0.36 | 42 | -0.23 | 0.817 |
| Genotype High-Affinity (vs. Mixed) | 1.48 | 0.31 | 42 | 4.77 | <.0001 |
| Healthy (vs. MDD) | -0.62 | 0.34 | 42 | -1.82 | 0.075 |
| **Anterior Cingulate Cortex (ACC)** |  |  |  |  |  |
| Intercept | 5.35 | 1.12 | 42 | 4.78 | <.0001 |
| Age | 0.03 | 0.01 | 42 | 1.93 | 0.061 |
| BMI | -0.15 | 0.03 | 42 | -4.39 | <.0001 |
| Non-White (vs. White) | 0.01 | 0.37 | 42 | 0.03 | 0.974 |
| Genotype High-Affinity (vs. Mixed) | 1.62 | 0.31 | 42 | 5.19 | <.0001 |
| Healthy (vs. MDD) | -0.67 | 0.34 | 42 | -1.97 | 0.056 |
| **Sensitivity: Secondary Analyses (No Additional Covariates** | | | | | |
| **Subgenual Prefontal Cortex (sgPFC)** |  |  |  |  |  |
| Intercept | 2.42 | 0.46 | 44 | 5.25 | <.0001 |
| Genotype High-Affinity (vs. Mixed) | 1.32 | 0.37 | 44 | 3.59 | 0.001 |
| Healthy (vs. Unmedicated MDD) | -0.66 | 0.44 | 44 | -1.49 | 0.142 |
| Medicated MDD (vs. Unmedicated MDD) | -0.01 | 0.47 | 44 | -0.02 | 0.984 |
| Healthy (vs. Medicated MDD) | -0.65 | 0.40 | 44 | -1.63 | 0.109 |
| **Anterior Cingulate Cortex (ACC)** |  |  |  |  |  |
| Intercept | 2.57 | 0.48 | 44 | 5.37 | <.0001 |
| Genotype High-Affinity (vs. Mixed) | 1.46 | 0.38 | 44 | 3.81 | 0.000 |
| Healthy (vs. Unmedicated MDD) | -0.68 | 0.46 | 44 | -1.48 | 0.145 |
| Medicated MDD (vs. Unmedicated MDD) | -0.06 | 0.48 | 44 | -0.12 | 0.905 |
| Healthy (vs. Medicated MDD) | -0.63 | 0.42 | 44 | -1.50 | 0.140 |
| **Sensitivity: Secondary Analyses (with additional covariates)** | | | | | |
| **Subgenual Prefontal Cortex (sgPFC)** |  |  |  |  |  |
| Intercept | 4.84 | 1.12 | 41 | 4.32 | <.0001 |
| Age | 0.03 | 0.02 | 41 | 2.00 | 0.052 |
| BMI | -0.13 | 0.03 | 41 | -3.90 | 0.000 |
| Non-White (vs. White) | -0.10 | 0.37 | 41 | -0.28 | 0.780 |
| Genotype High-Affinity (vs. Mixed) | 1.44 | 0.32 | 41 | 4.53 | <.0001 |
| Healthy (vs. Unmedicated MDD) | -0.73 | 0.39 | 41 | -1.88 | 0.068 |
| Medicated MDD (vs. Unmedicated MDD) | -0.26 | 0.43 | 41 | -0.61 | 0.547 |
| Healthy (vs. Medicated MDD) | -0.47 | 0.42 | 41 | -1.12 | 0.268 |
| **Anterior Cingulate Cortex (ACC)** |  |  |  |  |  |
| Intercept | 5.37 | 1.13 | 41 | 4.76 | <.0001 |
| Age | 0.03 | 0.02 | 41 | 2.02 | 0.050 |
| BMI | -0.14 | 0.03 | 41 | -4.33 | <.0001 |
| Non-White (vs. White) | -0.01 | 0.37 | 41 | -0.02 | 0.981 |
| Genotype High-Affinity (vs. Mixed) | 1.58 | 0.32 | 41 | 4.93 | <.0001 |
| Healthy (vs. Unmedicated MDD) | -0.80 | 0.39 | 41 | -2.03 | 0.049 |
| Medicated MDD (vs. Unmedicated MDD) | -0.29 | 0.44 | 41 | -0.66 | 0.511 |
| Healthy (vs. Medicated MDD) | -0.51 | 0.42 | 41 | -1.21 | 0.235 |

**Note:** Sensitivity analyses comparing TSPO binding (***V*_T_**) in models as described in Tables S1 and S2.
